# Rapamycin Promotes ROS-Mediated Cell Death *via* Functional Inhibition of xCT Expression in Melanoma Under γ-Irradiation

**DOI:** 10.3389/fonc.2021.665420

**Published:** 2021-04-20

**Authors:** Yunseo Woo, Hyo-Ji Lee, Jeongyeon Kim, Seung Goo Kang, Sungjin Moon, Jeong A. Han, Young Mee Jung, Yu-Jin Jung

**Affiliations:** ^1^ Department of Biological Sciences, Kangwon National University, Chuncheon, South Korea; ^2^ Kangwon Radiation Convergence Research Support Center, Kangwon National University, Chuncheon, South Korea; ^3^ Graduate Program in BIT Medical Convergence, Kangwon National University, Chuncheon, South Korea; ^4^ Department of Systems Immunology, Kangwon National University, Chuncheon, South Korea; ^5^ Department of Biochemistry and Molecular Biology, Kangwon National University, Chuncheon, South Korea; ^6^ Department of Chemistry, Kangwon National University, Chuncheon, South Korea

**Keywords:** melanoma, macrophage, YM1, KEAP1, NRF2, mTOR, radiation

## Abstract

Although many cancer patients are administered radiotherapy for their treatment, the interaction between tumor cells and macrophages in the tumor microenvironment attenuates the curative effects of radiotherapy. The enhanced activation of mTOR signaling in the tumors promotes tumor radioresistance. In this study, the effects of rapamycin on the interaction between tumor cells and macrophages were investigated. Rapamycin and 3BDO were used to regulate the mTOR pathway. *In vitro*, tumor cells cocultured with macrophages in the presence of each drug under normoxic or hypoxic conditions were irradiated with γ–rays. *In vivo*, mice were irradiated with γ–radiation after injection with DMSO, rapamycin and 3BDO into tumoral regions. Rapamycin reduced the secretion of IL-4 in tumor cells as well as YM1 in macrophages. Mouse recombinant YM1 decreased the enhanced level of ROS and the colocalized proportion of both xCT and EEA1 in irradiated tumor cells. Human recombinant YKL39 also induced results similar to those of YM1. Moreover, the colocalized proportion of both xCT and LC3 in tumor tissues was elevated by the injection of rapamycin into tumoral regions. Overall, the suppression of mTOR signaling in the tumor microenvironment might be useful for the improvement of tumor radioresistance.

## Introduction

Radiation therapy aims to kill cancer cells through direct damage to DNA and indirect reactive oxygen species (ROS)-induced damage to cellular organelles (1, 2). However, since tumor resistance to γ-radiation is observed in various tumor types, radioresistance factors should be considered to ensure effective radiotherapy (3). In particular, the crosstalk between tumor cells and macrophages in the tumor microenvironment has been regarded as an important factor among the tumor radioresistance factors that make radiotherapy challenging (4). Macrophages can be polarized into antitumoral M1 or protumoral M2 populations depending on their microenvironment (5). Unlike the M1 phenotype, the M2 phenotype has been reported to have resistance against γ-irradiation (6). Therefore, functional inhibition of M2 macrophages in the tumor microenvironment may be an alternative strategy for effective radiotherapy (7).

Tumor-secreted factors, such as IL-4, IL-10, and M-CSF, can induce the polarization of macrophages into M2-like tumor-associated macrophages (M2-like TAMs) (8, 9). Among these factors, IL-4 is a typical TH2 cytokine that promotes the expression of YM1, arginase-1, PPARγ, Fizz1 and VEGF in macrophages (10). IL-4 is found in various melanoma cells, including the human SK-MEL-28 and mouse B16F10 cell lines (11). Thus, it should be investigated whether M2-like TAMs induced by IL-4 released from tumor cells enhance tumor radioresistance. YM1 is a chitinase-like protein (CLP) found in mammals (12). Most CLPs can be combined with chitin, but they are unable to decompose chitin due to a lack of chitinolytic enzymatic activity (13). Increased levels of CLPs in a host are relevant to TH2-related diseases (14). Chitinase 3-like 1 (CHI3L1, YKL40/BRP39) has been reported to accelerate the recruitment of macrophages into tumor tissues and to promote tumor angiogenesis in colorectal patients (15). CHI3L1 is also known to promote the invasion and migration of non-small cell lung cancer *via* activation of mTOR signaling (16). However, there has been little research on YM1, which is a CLP expressed only in mice.

Ionizing radiation can cause cell death in tumors through ROS-induced damage (17). However, a high antioxidant capacity is observed in various cancer types, which in tumors can be an obstacle to efficient radiotherapy (18). The mechanism of glutathione (GSH) synthesis is one of the antioxidant pathways in tumors (19). The heterodimeric glutamate-cystine transporter composed of SLC7A11 (xCT) and CD98 mediates the import of extracellular cystine into the cytoplasm during GSH biosynthesis (20). xCT expression can be controlled by the KEAP1-NRF2 pathway, and the transcriptional activity of NRF2 can depend on mTOR signaling (21). However, little is known regarding the direct control of xCT expression by mTOR signaling in tumors under γ-irradiation. In many tumor studies, inhibition of mTOR signaling has been shown to improve the efficacy of radiotherapy (22, 23). Therefore, the inhibitory effects of mTOR signaling on tumor radioresistance induced by the interaction between macrophages and tumor cells were investigated in the B16F10-derived microenvironment during γ-irradiation.

## Materials and Methods

### Cell Lines and BMDM Differentiation

All cell lines were obtained from the Korean Cell Line Bank (KCLB). The following cell lines were used in this study: B16F10 (KCLB Cat# 80008, Seoul, Republic of Korea) and L929 (KCLB Cat# 10001, Seoul, Republic of Korea). B16F10 cells were cultured with DMEM supplemented with 10% FBS and 1% penicillin/streptomycin in cell culture plates at 37°C and 5% CO_2_ until confluent (~90%) (24). L929 cells were maintained in complete RPMI medium for 5 days before harvesting cell supernatants (25). The supernatant, which was used as a source of M-CSF, was filtered through a 0.2-μm filter and then aliquoted for storage at -20°C until use. To differentiate BMDMs, the tibia and femur of each hind leg were obtained from C57BL/6 mice (Nara Biotech, Seoul, Republic of Korea). The whole bone marrow was flushed out using a 1-ml syringe with a 26-G needle. The cells were cultured with DMEM containing 20% FBS and 30% L929-conditioned medium in 100-mm petri dishes for 5 days.

### Reagents

Rapamycin (914.18 g/mol) and 3BDO (327.33 g/mol) were purchased from Selleckchem (Houston, TX, USA). To control mTOR signaling, cells were treated with 100 nM rapamycin (mTOR inhibitor/autophagy activator) or 100 nM 3BDO (mTOR activator/autophagy inhibitor). DPI (10 μM; NADPH oxidase inhibitor; Sigma-Aldrich, St. Louis, MO, USA) was used to deplete intracellular ROS.

### Animal Studies

The animal experiments in the present study were approved and confirmed by the Ethical Guidelines for Animal Experiments of Kangwon National University (KW-181214-1). B16F10 cells (5 × 10^5^) were inoculated subcutaneously into C57BL/6 male mice (5–8 weeks old, n=5/group) (26). After 7 days, when palpable tumors (5 mm in diameter) developed, the mice were treated with intratumoral injections of DMSO, rapamycin (0.1 mg/kg/day) or 3BDO (0.036 mg/kg/day) on days 7-14. Whole-body irradiation was performed once on day 7 after drug injections at 3 h. All tumor samples were harvested on day 14. Tumor volumes were measured using calipers and calculated based on the ellipsoid formula (a × b × c × π × 4/3).

### *In Vitro* Coculture Experiments

To understand the radioresistance roles of macrophages in the tumor microenvironment, Transwell permeable supports (0.4-μm pore size, Costar 3450, Corning Inc., Kennebunk, ME, USA) were utilized. B16F10 cells (3 × 10^5^) were seeded in the bottom chamber, and bone marrow-derived macrophages (BMDMs) (1 × 10^5^) were plated in the upper chamber. Each cell type was incubated separately at 37°C and 5% CO_2_ for 24 h. After washing, the cells were cocultured under various experimental conditions.

### Hypoxic Incubation

A hypoxia chamber (PDS-1000, COY Laboratory Products, Inc., Grass Lake, MI, USA) was filled with a mixture of 95% N_2_ and 5% CO_2_ gas at 2 p.s.i. for 1 h. Cells were incubated at 37°C in a humidified atmosphere containing 1% O_2_ and 5% CO_2_ for 24 h.

### γ-Irradiation

To evaluate the effects of γ-radiation on the tumor microenvironment, each sample was irradiated with γ-rays using a Cs-137 source (Gammacell 40 Extractor, Best Theratronics Ltd., Ottawa, Canada). Cells were irradiated with 3 Gy *in vitro*, and mice were exposed to whole-body irradiation at doses of 0, 1, 3, 5, 7 or 9 Gy *in vivo*.

### Western Blot Analysis

B16F10 cells were homogenized with RIPA buffer supplemented with Halt protease and a phosphatase inhibitor cocktail (Thermo Scientific Pierce, Rockford, IL, USA). Samples were separated by SDS-PAGE (27). The membranes were incubated with specific primary and secondary antibodies, which were washed three times with TBS-T. The membranes that were activated using an ECL solution (Advansta, Menlo Park, CA, USA) were developed with AGFA X-ray film (Agfa-Gevaert NV, Mortsel, Belgium).

### Confocal Microscopy

Confocal microscopy was performed as previously described (28). Briefly, B16F10 cells were plated onto coverslips (1 × 10^5^ cells per coverslip; Paul Marienfeld GmbH & Co., KG, Lauda-Könighofen, Germany) in 12-well plates. The cells were treated with various stimuli as described in the figure legends. The cells were fixed with 4% paraformaldehyde and permeabilized with 0.2% Triton X-100 in PBS. After blocking, specific antibodies were used for intracellular staining. The samples were mounted and observed under a confocal microscope (FV1000; Olympus Corporation, Tokyo, Japan). The quantification of all images was performed by using ImageJ (version 1.48, National Institutes of Health, Bethesda, MD, USA).

### ELISA

ELISA was performed as previously described (29). IL-4 and YM1 levels were measured by using the Murine IL-4 Mini ABTS ELISA Development Kit (PeproTech EC Ltd, London, UK) and the Mouse YM1/Chitinase 3-like 3 DuoSet ELISA Development Kit (R&D Systems, Minneapolis, MN, USA) according to the instructions of the manufacturer.

### Determination of Cell Numbers

The number of viable cells was assessed by trypan blue exclusion assay. The trypan blue exclusion assay was performed as previously described (29). The determination of cell numbers was performed with the Countess Automated Cell Counter (Invitrogen, Carlsbad, CA, USA).

### Evaluation of GEO Profiles

The Gene Expression Omnibus (GEO) database was utilized in this study. GSE29074 (https://www.ncbi.nlm.nih.gov/geo/query/acc.cgi?acc=GSE29074) data sets were used to compare the expression values of IL-4, xCT, and YM1 in wild-type mouse tissues or in melanoma tissues derived from the iMet model (metastatic melanoma, Tyr-rtTA;Tet-Met;Ink4a/Arf^-/-^) and iHRAS model (non-metastatic melanoma, Tyr-rtTA;Tet-HRASV12G;Ink4a/Arf^-/-^) (30). For the human study, the expression values of IL-4, xCT, and YKL39 from 70 melanoma patients (7 normal skin, 18 benign skin nevi and 45 primary malignant melanoma profiles) were obtained from GSE3189 (https://www.ncbi.nlm.nih.gov/geo/query/acc.cgi?acc=GSE3189) (31). Gene expression values are presented in a scatter plot graph created with GraphPad Prism 5 software (GraphPad Software, Inc., La Jolla, CA, USA).

### Statistical Analysis

In this study, all data are presented as the mean ± standard deviation. Statistical analysis was performed with one-way or two-way analysis of variance (ANOVA) followed by the Bonferroni test to compare all pairs of columns (95% confidence intervals). Analyses were performed with GraphPad Prism 5. P values < 0.05 were considered significant. Experiments were repeated a minimum of 3 times for each condition.

## Results

### Rapamycin Promotes Radiation-Induced Cell Death in Mouse Melanoma *In Vivo*

**Figure 1:**
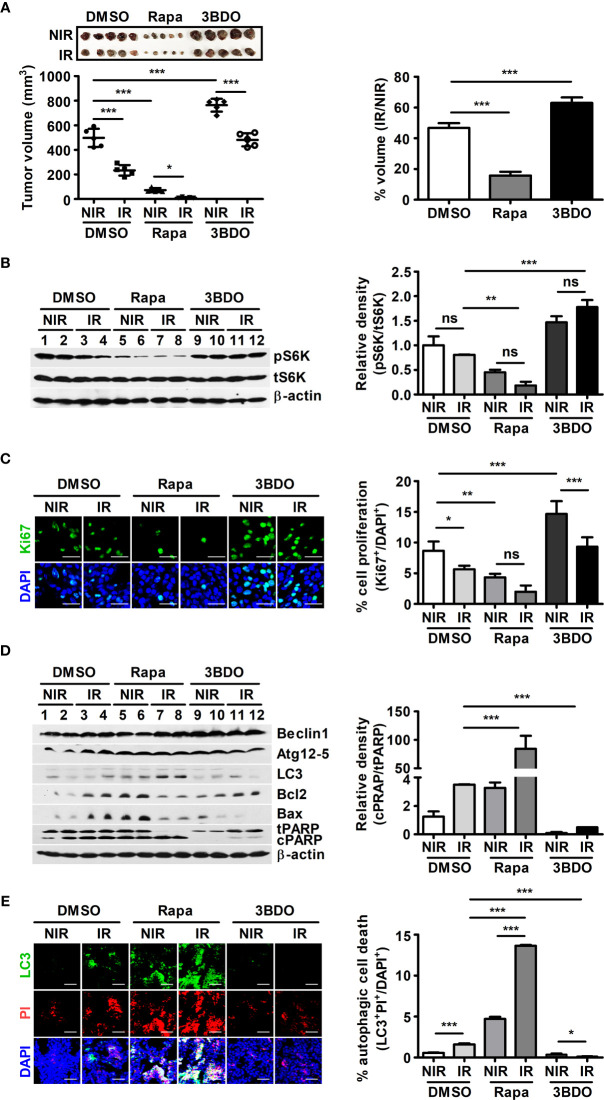
Pharmaceutical regulation of mTOR activation affects B16F10-tumor development. **(A)** After tumor resection, all tumors were measured with calipers to calculate tumor volumes (left). To evaluate tumor radiosensitivity, percentages were calculated by dividing the volume of irradiated tumors (IR) by the volume of nonirradiated tumors (NIR) in each group (right). **(B)** The protein levels of p70 S6K, phospho-p70 S6K, and β-actin in homogenized tumor tissue samples were evaluated by western blotting. The relative density of phosphorylated forms was measured using ImageJ. **(C)** Intratumoral levels of Ki67 were analyzed by confocal microscopy. Nuclei (blue) and proliferating cells (green) were stained with DAPI and an anti-Ki67 antibody, respectively. Scale bars, 30 μm. **(D)** The protein levels of Beclin1, Atg12-5, LC3, Bcl2, Bax, PARP, and β-actin in homogenized tumor tissue samples were evaluated by western blotting. **(E)** Intratumoral levels of LC3 (green) were assessed by confocal microscopy. Nuclei (blue) and damaged cells (red) were stained with DAPI and propidium iodide, respectively. Scale bars, 100 μm. The bars and error bars represent the mean ± SD; *P < 0.05; **P < 0.01; ***P < 0.001; ns, not significant.

In radiotherapy, it is important to determine the minimum dose that delivers maximum energy to cancer cells and causes minimal damage to surrounding healthy tissues (32). B16F10 tumor-bearing mice were irradiated with γ-radiation to determine the minimum dose that could significantly affect tumor volume, and a 3-Gy dose was selected (**Supplementary Figures 1A, B**). To elucidate the roles of mTOR signaling in the tumor microenvironment during radiotherapy, rapamycin and 3BDO were used to pharmaceutically control mTOR activation in the B16F10 tumor model (**Supplementary Figure 1C**). Compared with DMSO, rapamycin reduced tumor size, whereas 3BDO increased the volume of tumors (**Supplementary Figure 1D** and **Figure 1A**). Rapamycin also promoted a reductive effect of γ-radiation on tumors (**Figure 1A**). Rapamycin and 3BDO, as pharmaceutical regulators of mTOR activation, worked effectively in tumor tissues (**Figure 1B**). These results suggest that pharmaceutical control of mTOR signaling affects tumor development and efficient radiotherapy. Tumor development is closely related to tumor proliferation and cell death in the tumor microenvironment (33). Rapamycin reduced the proportion of Ki67^+^ cells in tumor tissues (**Figure 1C**). To determine the relationship between the difference in tumor volume and the cell death mechanism related to the mTOR pathway, the expression levels of autophagy-related (Beclin1, Atg12-5 complex, and LC3) or apoptosis-related proteins (Bcl-2, Bax, and PARP) were examined (**Figure 1D**). Compared to other groups, enhanced LC3 expression was detected in rapamycin-injected tumor tissues, and the expression levels of other autophagy-related genes, Beclin1 and Atg12-5 complex, were not changed significantly. The expression levels of Bcl2 and Bax were also enhanced in rapamycin-treated tissues, however, γ-radiation reduced the expression levels of those genes. Rapamycin also increased the ratio of cleaved PARP (cPARP) to total PARP (tPARP) in nonirradiated tumor tissues and promoted this ratio in irradiated tumors even further than other groups (**Figure 1D**). To clarify the types of regulated cell death leading to PARP cleavage in tumor tissues, autophagic (LC3^+^PI^+^) and apoptotic (Caspase-3^+^PI^+^) cell death was evaluated using confocal microscopy. Under γ-irradiation, the proportion of LC3^+^PI^+^ cells was significantly increased in rapamycin-injected tissues compared to other groups (**Figure 1E**). However, the patterns of apoptotic cell death induced by drugs and radiation were shown to be different from the tumor development patterns in **Figure 1A** (**Supplementary Figure 1E**). Overall, the pharmaceutical inhibition of mTOR signaling by rapamycin not only inhibits tumor development but also promotes the reductive effect of γ-radiation on tumor volumes.

### Rapamycin Promotes Radiation-Induced Cell Death in Melanoma Cells Cocultured With Macrophages *In Vitro*

**Figure 2:**
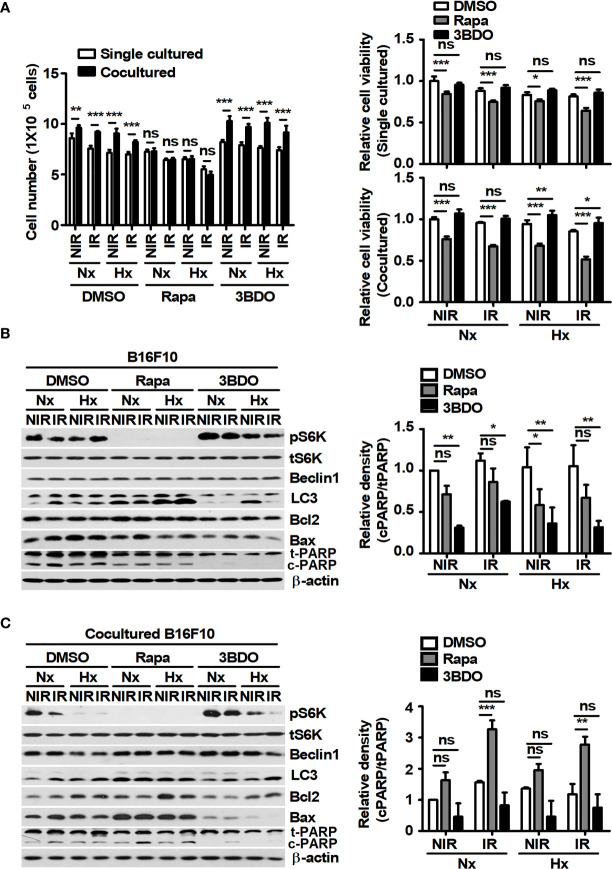
Pharmaceutical regulation of mTOR activation affects macrophage-mediated B16F10 cell survival. B16F10 cells cultured alone or cocultured with BMDMs were incubated in the presence of 100 nM rapamycin or 100 nM 3BDO at 37°C/5% CO_2_ in a normoxic (Nx) chamber or 37°C/1% O_2_ in a hypoxic (Hx) chamber for 3 h. After 3-Gy γ-irradiation, B16F10 cells were incubated under the indicated conditions for 21 h. **(A)** Cell viability was measured by an automated cell counter. **(B, C)** The protein levels of p70 S6K, phospho-p70 S6K, Beclin1, LC3, Bcl2, Bax, and PARP in B16F10 cells were evaluated by western blotting. β-actin was used as an internal control. The band density of each sample was measured with ImageJ. The bars and error bars represent the mean ± SD; *P < 0.05; **P < 0.01; ***P < 0.001; ns, not significant.

Macrophages in the tumor microenvironment have been reported to promote tumor development and contribute to tumor radioresistance (34). Thus, an *in vitro* coculture system was established to investigate whether macrophages in the tumor microenvironment affect the viability of tumor cells under γ-radiation during the pharmaceutical control of mTOR signaling. In addition, various oxygen conditions, such as normoxia and hypoxia, were introduced to mimic the tumor microenvironment (35). The viability of B16F10 cells cocultured with BMDMs was higher than that of single-cultured B16F10 cells, excluding the rapamycin-treated groups (**Figure 2A**). Compared to both the DMSO- and 3BDO-groups in single culture conditions, rapamycin reduced the viability of single-cultured-B16F10 cells, regardless of oxygen and radiation conditions (**Figure 2A**). These results indicate that the inhibitory effect of rapamycin on mTOR signaling could not only directly decrease the viability of B16F10 cells but also attenuate the macrophage-mediated increase in B16F10 cell viability. In **Figure 2A**, because rapamycin affected the cell viability of B16F10 cells under various culture conditions, the ratio of cPARP/tPARP was assessed in B16F10 cells under the same conditions. As a result, the effects of mTOR inhibition by rapamycin on the cleavage patterns of PARP in B16F10 cells were different between single and coculture conditions (**Figures 2B, C**). In other words, rapamycin increased the cleaved levels of PARP in B16F10 cells cocultured with BMDMs relative to those in tumor tissues, regardless of oxygen conditions during irradiation. Overall, these results indicate that macrophages may play beneficial roles in tumor survival under γ-irradiation and that rapamycin can interfere with the interaction between macrophages and tumor cells in the tumor microenvironment.

### Rapamycin Attenuates the Expression and Secretion of YM1 in Macrophages Cocultured With Melanoma Cells

**Figure 3:**
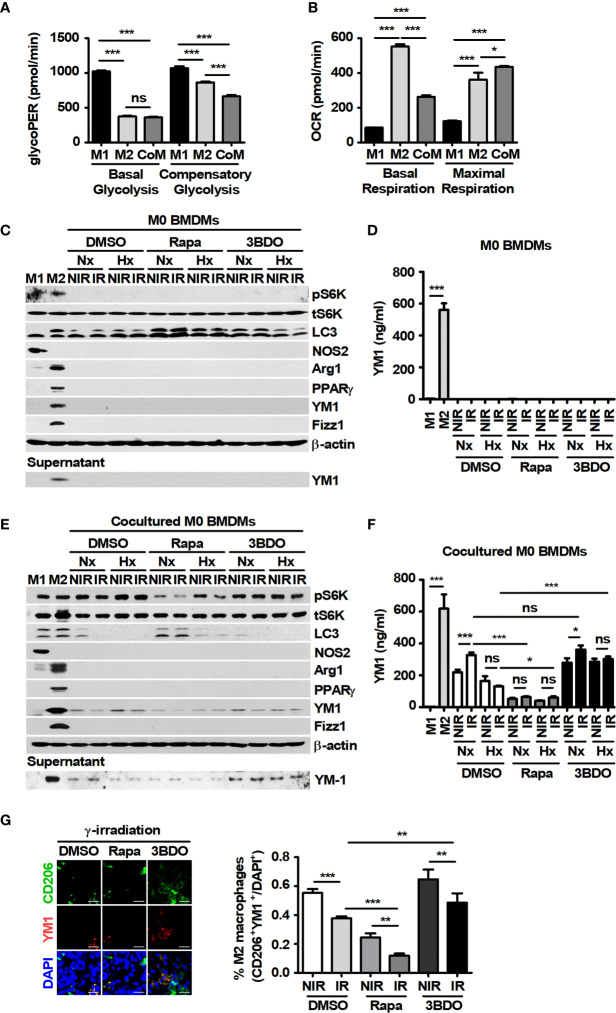
Pharmaceutical regulation of mTOR activation affects YM1-expressing macrophages cocultured with B16F10 cells. **(A, B)** BMDMs were treated with 100 ng/ml LPS plus 20 ng/ml IFN-γ or 20 ng/ml IL-4, or were cocultured with B16F10 cells for 24 h, and then the glycolytic proton efflux rate (GlycoPER) **(A)** or the oxygen consumption rate (OCR) **(B)** in the cells was analyzed. **(C–F)** BMDMs were polarized into the M1 (LPS+IFN-γ) and M2 (IL-4) phenotypes. M1-, M2-, M0- and B16F10-cocultured BMDMs were incubated under the indicated conditions. The protein levels of p70 S6K, phospho-p70 S6K, LC3, NOS2, Arginase-1, PPAR-γ, YM-1, Fizz-1, and β-actin in BMDMs were evaluated by western blotting. Secreted YM-1 in the supernatant was detected by western blot and ELISA. **(G)** Intratumoral levels of CD206 and YM-1 were analyzed by confocal microscopy. CD206^+^YM1^+^ cells in tissue samples were assessed by ImageJ. The bars and error bars represent the mean ± SD; *P < 0.05; **P < 0.01; ***P < 0.001; ns, not significant.

In the tumor microenvironment, antitumoral M1-like TAMs and protumoral M2-like TAMs are distributed according to the surrounding environmental conditions (36). First of all, the effects of tumor cells on macrophages were investigated because tumor-secreted factors can induce the polarization of macrophages. The changes in polarization markers were evaluated in BMDMs under the indicated conditions (**Supplementary Figure 2** and **Figure 3**). M1 macrophages have been reported to express *nos2 and cxcl10;* otherwise, M2 macrophages express *fizz1, arg1, pparγ*, and *ccl22*. LPS plus INF-γ induced the polarization of M0 to M1 macrophages, and IL-4 induced polarization to the M2 types (**Supplementary Figures 2A, B**). In addition, unique metabolic patterns were observed in M1 and M2 macrophages (**Supplementary Figures 2C, D**), and these results were consistent with those of a previous study (37). In M1-polarized cells, compared with DMSO treatment, rapamycin inhibited the attenuation of NOS2 expression after γ-radiation under normoxia and inhibited the increase in NOS2 expression after γ-radiation in the cells under hypoxia (**Supplementary Figure 2E**). However, 3BDO decreased the expression of NOS2 in M1 BMDMs under all conditions (**Supplementary Figure 2E**). In M2-polarized cells, compared with DMSO treatment, rapamycin attenuated the expression of M2 markers, excluding Fizz1, under all conditions (**Supplementary Figure 2F**). These results suggest that the pharmaceutical inhibition of mTOR signaling by rapamycin might attenuate the polarization of macrophages into the M2 phenotype. Meanwhile, because tumor-secreted factors could also stimulate the polarization of macrophages into an M2-like phenotype in the tumor microenvironment (38), the polarized characteristics of macrophages were investigated in BMDMs cocultured with B16F10 cells. In BMDMs cocultured with B16F10 cells, the glycolytic proton efflux rate (glycoPER) and the oxygen consumption rate (OCR) were similar to those in M2-BMDMs (**Figures 3A, B**). Although marker proteins of polarized macrophages were not detected in single-cultured BMDMs, the expression and secretion of YM1 were observed in BMDMs cocultured with B16F10 cells (**Figures 3C-F**). In addition, rapamycin attenuated the expression and secretion of YM1 in the cells (**Figures 3E, F**). To clarify the source of released YM1, the expression of YM1 was analyzed in B16F10 cells. The results showed that YM1 was not derived from B16F10 cells (**Supplementary Figures 2G, H**). Among the M2-related proteins, YM1 might be specifically expressed in macrophages stimulated by TH2 cytokines (39). To identify the prominent expression of YM1 in BMDMs cocultured with B16F10 cells, BMDMs were treated with various concentrations of IL-4. Only YM1 was expressed in BMDMs stimulated with 0.02 ng/ml IL-4 (**Supplementary Figure 2I**). These results *in vitro* were also observed *in vivo*. The proportion of CD206^+^YM1^+^ cells was reduced by rapamycin injection and was decreased by γ-irradiation even further than those in the other groups (**Figure 3G**). Overall, rapamycin attenuated the polarization of macrophages by B16F10 into YM1-producing cells with M2-like metabolism.

### Rapamycin Attenuates the Expression and Secretion of IL-4 in Tumor Cells

**Figure 4:**
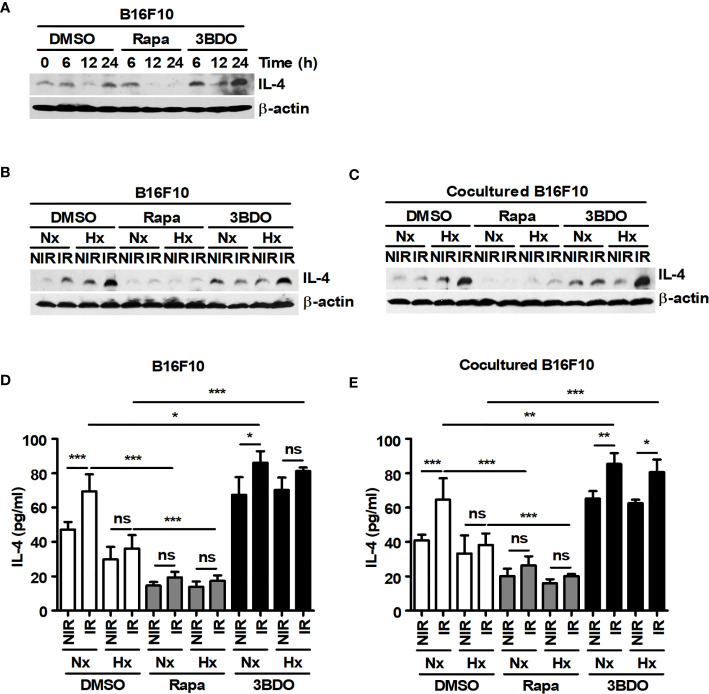
Pharmaceutical regulation of mTOR activation affects the expression and secretion of IL-4 in B16F10 cells. **(A)** B16F10 cells were treated with DMEM containing DMSO, 100 nM rapamycin or 100 nM 3BDO for the indicated times. Intracellular IL-4 levels were detected by western blotting. **(B–E)** B16F10 cells cultured alone or cocultured with BMDMs were incubated in the presence of 100 nM rapamycin or 100 nM 3BDO under the indicated conditions for 21 h. Intracellular IL-4 levels were detected by western blotting **(B, C),** and secreted IL-4 was analyzed by ELISA **(D, E)**. The bars and error bars represent the mean ± SD; *P < 0.05; **P < 0.01; ***P < 0.001; ns, not significant.

The production of YM1 in macrophages can be induced by TH2 cytokines, such as IL-4 and IL-13 (40). In previous studies, IL-4 has been reported to be produced in various melanoma cells, including B16F10 cells (11, 41). Therefore, IL-4 expression was investigated in B16F10 cells. B16F10 cells showed considerable expression of IL-4 at the basal level, and its expression was attenuated by rapamycin (**Figure 4A**). Although various culture conditions, such as irradiation and oxygen status, affected the expression and secretion of IL-4 in B16F10 cells, rapamycin significantly attenuated the levels of IL-4 production in the cells (**Figures 4B**–**E**). In addition, the expression and secretion of IL-4 in B16F10 cells under various culture conditions were independent of the presence or absence of macrophages (**Figures 4B**–**E**). These results suggest that IL-4 released by tumor cells may be an important factor for skewing macrophages toward the M2 population and that mTOR inhibitors may be effective drugs for blocking IL-4-mediated signaling in the tumor microenvironment.

### YM1 Rescues the Decreased Viability of Melanoma *via* Attenuation of Radiation-Induced ROS

**Figure 5:**
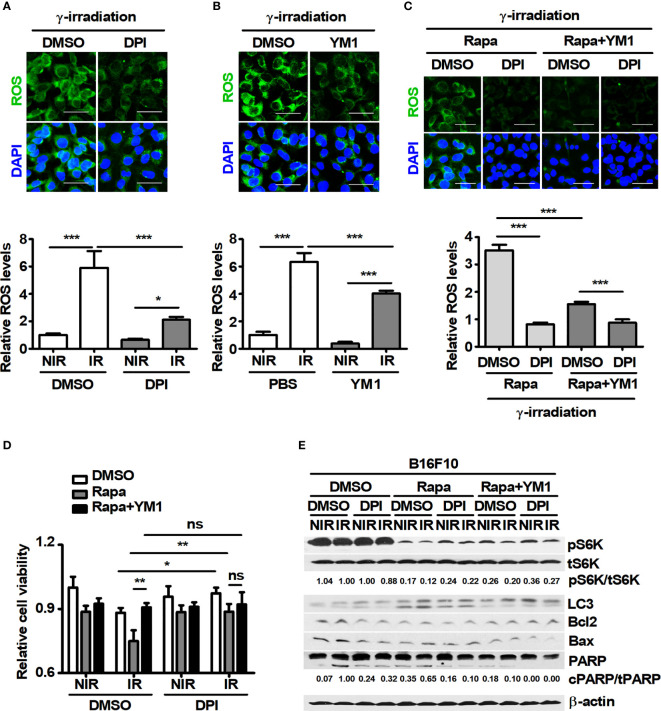
YM1 affects ROS-induced cell death in B16F10 cells under γ-radiation. **(A–E)** After pretreatment with diphenyleneiodonium (DPI) for 1 h, B16F10 cells were treated with DMSO or 100 nM rapamycin for 3 h. B16F10 cells were washed with PBS and then incubated with DMEM and rapamycin in the presence or absence of 125 ng/ml rmYM-1 for 3 h before receiving 3-Gy γ-irradiation. After γ-irradiation, B16F10 cells were incubated with 5 μM CellROX for 30 min. **(A–C)** Intracellular ROS were assessed by confocal microscopy. Scale bars, 50 μm. **(D)** Cell viability was measured by an automated cell counter. **(E)** The protein levels of p70 S6K, phospho-p70 S6K, LC3, Bcl2, Bax, and PARP in B16F10 cells were evaluated by western blotting. β-actin was used as an internal control. The bars and error bars represent the mean ± SD; *P < 0.05; **P < 0.01; ***P < 0.001; ns, not significant.

A large amount of intracellular ROS induced by γ-radiation could contribute to cell death in tumors (42). However, various cancer cells might be resistant to γ-ray-induced ROS because of high levels of antioxidant mechanisms in the cells (43). Therefore, the levels of intracellular ROS induced by a 3-Gy-dose of γ-irradiation were investigated in B16F10 cells. In addition, since B16F10 induced YM1 expression in BMDMs during coculture, the effect of recombinant mouse YM1 on ROS levels was also analyzed in B16F10 cells. 3-Gy-irradiation induced intracellular ROS in B16F10 cells and the levels of γ-ray-induced ROS were attenuated by pretreatment with diphenyleneiodonium chloride (DPI), known as a ROS scavenger (**Figures 5A, C**). Recombinant YM1 diminished the levels of γ-ray-induced ROS not only in DMSO-B16F10 cells but also in rapamycin-B16F10 cells (**Figures 5B, C**). Since recombinant YM1 attenuated ROS levels in γ-irradiated B16F10 cells, the effect of YM1 on the cell viability of B16F10 cells was investigated. As a result, DPI restored the reduced viability of B16F10 cells treated with 3 Gy irradiation in DMSO- and rapamycin-B16F10 cells (**Figure 5D**). DPI also decreased the ratio of cPARP/tPARP promoted by irradiation in the same groups (**Figure 5E**). This means that a 3-Gy-dose of γ-irradiation could induce the death of B16F10 cells through the generation of intracellular ROS. Meanwhile, recombinant YM1 rescued the cell viability and reduced the increased ratio of cPARP/tPARP in the rapamycin groups during irradiation (**Figures 5D, E**). Overall, these results suggest that inhibition of mTOR signaling in tumor cells may promote their sensitivity to γ-ray-induced ROS.

### Rapamycin Attenuates the Functional Expression of YM1-Induced xCT in Melanoma Cells

**Figure 6:**
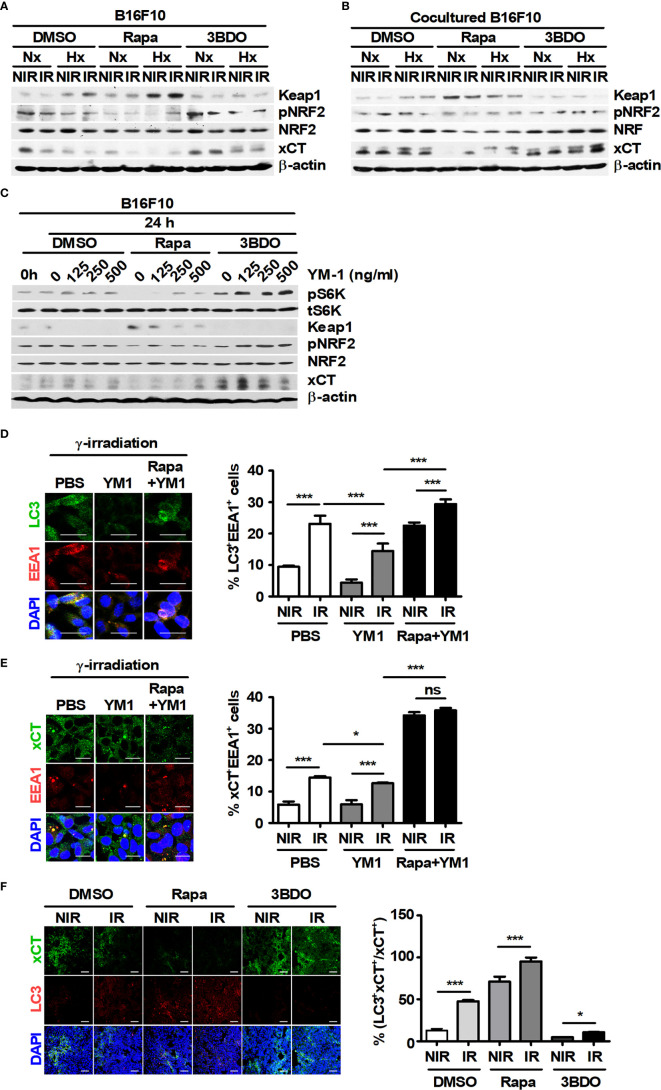
Pharmaceutical regulation of mTOR activation affects the functional expression of xCT in B16F10 cells. **(A, B)** B16F10 cells cultured alone or cocultured with BMDMs were treated with DMSO, 100 nM rapamycin or 100 nM 3BDO under the indicated conditions. Intracellular levels of Keap1, phospho-NRF2, NRF, xCT, and β-actin in B16F10 cells were evaluated by western blotting. **(C–E)** B16F10 cells were treated with DMSO, 100 nM rapamycin or 100 nM 3BDO for 3 h and then stimulated with the indicated concentrations of recombinant mouse YM-1 for 21 h. **(C)** The expression levels of phospho-p70 S6K, p70 S6K, Keap1, phospho-NRF2, NRF, xCT, and β-actin in B16F10 cells were detected by western blotting. **(D, E)** Intracellular levels of LC3, EEA1, and xCT were assessed by confocal microscopy. Scale bars, 30 μm. **(F)** Intratumoral levels of xCT and LC3 were analyzed by confocal microscopy. All images were quantified using ImageJ. Scale bars, 200 μm. The bars and error bars represent the mean ± SD; *P < 0.05; ***P < 0.001; ns, not significant.

The intracellular ROS in tumor cells can be regulated by the xCT system, a major pathway of GSH synthesis (44). Therefore, the effect of YM1 on xCT expression was investigated because recombinant YM1 attenuated the levels of ROS in B16F10 cells during irradiation. Compared with xCT expression in single cultured DMSO-B16F10 cells, BMDMs continued to induce its expression in DMSO-B16F10 cells under various stimuli during coculture (**Figures 6A, B**). Rapamycin attenuated xCT expression in B16F10 cells under both single- and cocultured conditions but not in other groups (**Figures 6A, B**). Recombinant YM1 stimulated mTOR signaling and also promoted xCT expression in B16F10 cells (**Figure 6C**). Rapamycin can promote EEA1 expression and induce the formation of amphisomes through the fusion of autophagosomes with early endosomes (45). The degradation of CD98, a component protein in the xCT system, can be induced by EEA1-mediated capture during its recycling process (46). Therefore, the effects of recombinant YM1 on the colocalization of LC3 and EEA1 (or EEA1 and xCT) were assessed in B16F10 cells. Compared to the nonirradiated groups, γ-radiation increased the proportions of LC3^+^EEA1^+^ and xCT^+^EEA1^+^ cells (**Figures 6D, E**). On the other hand, YM1 significantly reduced the proportions of LC3^+^EEA1^+^ and xCT^+^EEA1^+^ cells in the irradiated groups (**Figures 6D, E**). Rapamycin enhanced the proportions of LC3^+^EEA1^+^ and xCT^+^EEA1^+^ cells in the YM1-treated groups (**Figures 6D, E**).

**Figure 7:**
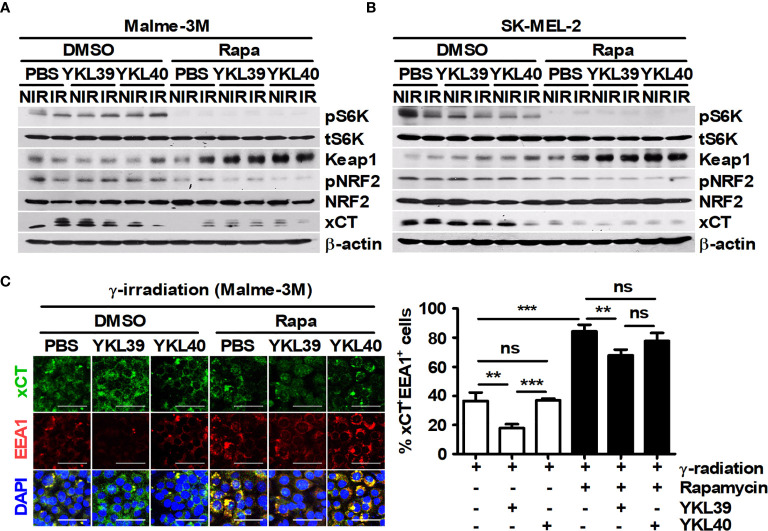
Human YKL39 affects γ-ray-induced colocalization of xCT and EEA1 in Malme-3M, a human melanoma cell line. **(A–C)** Malme-3M and SK-MEL-2 cells were treated with DMSO or 100 nM rapamycin for 3 h. After 3-Gy γ-irradiation, each cell line was stimulated with recombinant human YKL39 and YKL40 for 21 h. **(A, B)** The expression levels of phospho-p70 S6K, p70 S6K, Keap1, phospho-NRF2, NRF, xCT, and β-actin in the cells were detected by western blotting. **(C)** Intracellular levels of xCT and EEA1 were assessed by confocal microscopy. Scale bars, 50 μm. The bars and error bars represent the mean ± SD; **P < 0.01; ***P < 0.001; ns, not significant.

As mentioned in **Figure 1E**, rapamycin enhanced the proportion of LC3^+^PI^+^ cells in tumor tissues. Therefore, the relationship between LC3^+^ dots induced by rapamycin and xCT expression in tumor tissues was analyzed. Rapamycin decreased the proportion of xCT^+^ cells but increased the proportion of LC3^+^xCT^+^ cells in tumor tissues (**Figure 6F**). The effect of YM1 on the expression of metastasis-related proteins in B16F10 cells was investigated. As a result, rapamycin attenuated the expression of MMP2 and MMP9, but the effects of macrophages on their expression were not observed in B16F10 cells under coculture conditions compared with those in single-culture conditions (**Supplementary Figures 3A, B**). In addition, YM1 also failed to affect the expression of metastasis-related proteins (**Supplementary Figure 3C**). These results mean that YM1 might not be involved in melanoma metastasis. Overall, however, these results suggested that YM1 produced by macrophages may contribute to promoting xCT expression and attenuate the EEA1-mediated capture of intracellular xCT in tumor cells during irradiation. Meanwhile, among human CLPs, YKL39 and YKL40 can promote tumor development (47, 48). To investigate the effect of CLPs on xCT expression in human melanoma cell lines, Malme-3M and SK-MEL-2 cells were treated with YKL39 and YKL40 (**Figures 7A–C**). YKL39 increased the expression of xCT in nonirradiated Malme-3M cells (**Figure 7A**). Although YKL39 did not affect the expression of xCT in γ-irradiated Malme-3M cells, it decreased the proportion of xCT^+^EEA1^+^ cells regardless of rapamycin treatment under γ-radiation (**Figures 7A, C**). The effect of YKL40 on the expression of xCT was not observed in Malme-3M cells (**Figure 7A**). Meanwhile, YKL39 and YKL40 did not affect the expression of xCT in SK-MEL-2 cells (**Figure 7B**).

Finally, the expression values of IL-4, xCT, and YM1 in control or melanoma tissues derived from metastatic (iMet model) and non-metastatic (iHRAS model) tissues were compared using Gene Expression Omnibus (GEO) data (GSE29074). Only mRNA of YM1 was significantly increased in both melanoma tissues compared with that in normal tissues (**Supplementary Figures 4A**–**C**). To evaluate the associations between tumorigenesis and target genes (IL-4, xCT, and YKL39) in melanoma patients, clinical data in GEO profiles (GSE3189) were used. As a result, only xCT expression was significantly associated with tumor progression (**Supplementary Figures 4D**–**F**). These results imply that xCT could be closely associated with melanoma malignancy in humans. The cBioPortal database (Skin Cutaneous Melanoma, TCGA, PanCancer Atlas, 448 samples) was used to analyze the correlation between xCT expression and selected genes (KEAP1, NRF2, and YKL39) in melanoma patients. Although trends of negative (KEAP1) and positive (NRF2 and YKL39) regression lines were observed, they did not appear to show a correlation between xCT and each gene (**Supplementary Figures 4G**–**I**).

## Discussion

It is important to understand the interaction between macrophages and tumor cells in the tumor microenvironment as a strategy to increase the efficiency of cancer radiotherapy (3). Moreover, finding a radiotherapy adjuvant that can reduce side effects in hosts receiving γ-irradiation is crucial. Since the contribution of mTOR signaling to tumorigenesis has been revealed in many tumor studies, targeting mTOR as a radiosensitizing strategy may be a reasonable approach.

Rapamycin is a widely used drug that can effectively block the mTOR pathway (49). Rapamycin works as an allosteric inhibitor of the FRB domain of mTORC1 through the formation of a complex with FKBP12 (also known as FKBP 1A), and it can also activate autophagy (50). 3BDO can directly activate mTOR *via* hydrogen bonding to FKBP1A and can be used as an autophagy inhibitor (51). Therefore, these two agents are suitable drugs to study the pharmaceutical effects of mTOR control on the tumor microenvironment because they can cause conflicting results by targeting the same protein. In this study, the effects of these drugs on the interaction between macrophages and tumor cells during irradiation were evaluated.

Inhibiting antioxidant activity in tumor cells during radiotherapy can be a major therapeutic strategy for increasing radiosensitivity (18). In this study, the reduction in xCT expression by mTOR inhibition was observed. In addition, the EEA1-mediated capture of intracellular xCT was newly discovered. The regulation of xCT activity in tumor cells *via* mTOR inhibition in this study can be supported by recently published studies (52, 53). Therefore, inhibiting functional xCT in tumors through the suppression of mTOR signaling might be an alternative strategy for efficient radiotherapy (21).

In this study, experiments using neutralizing antibodies against IL-4 and YM1 were not performed both *in vitro* and *in vivo*. These experimental approaches are essential for understanding the interaction between macrophages and tumor cells in the tumor microenvironment. However, the contribution of the interaction between the cells to tumor radioresistance was indirectly confirmed through recombinant proteins, such as IL-4 and YM1.

YM1 produced by macrophages may promote the expression of functional xCT *via* mTOR signaling in melanoma while mTOR inhibitors, including rapamycin, may promote the sensitivity of tumor cells to γ-ray-induced ROS *via* the functional attenuation of xCT in tumors. Although various culture conditions were utilized *in vitro* to mimic the tumor microenvironment, several ambiguous results, such as the levels of phosphorylated S6K, were observed among various conditions. These unexpected results should be clarified through further studies.

**Figure 8:**
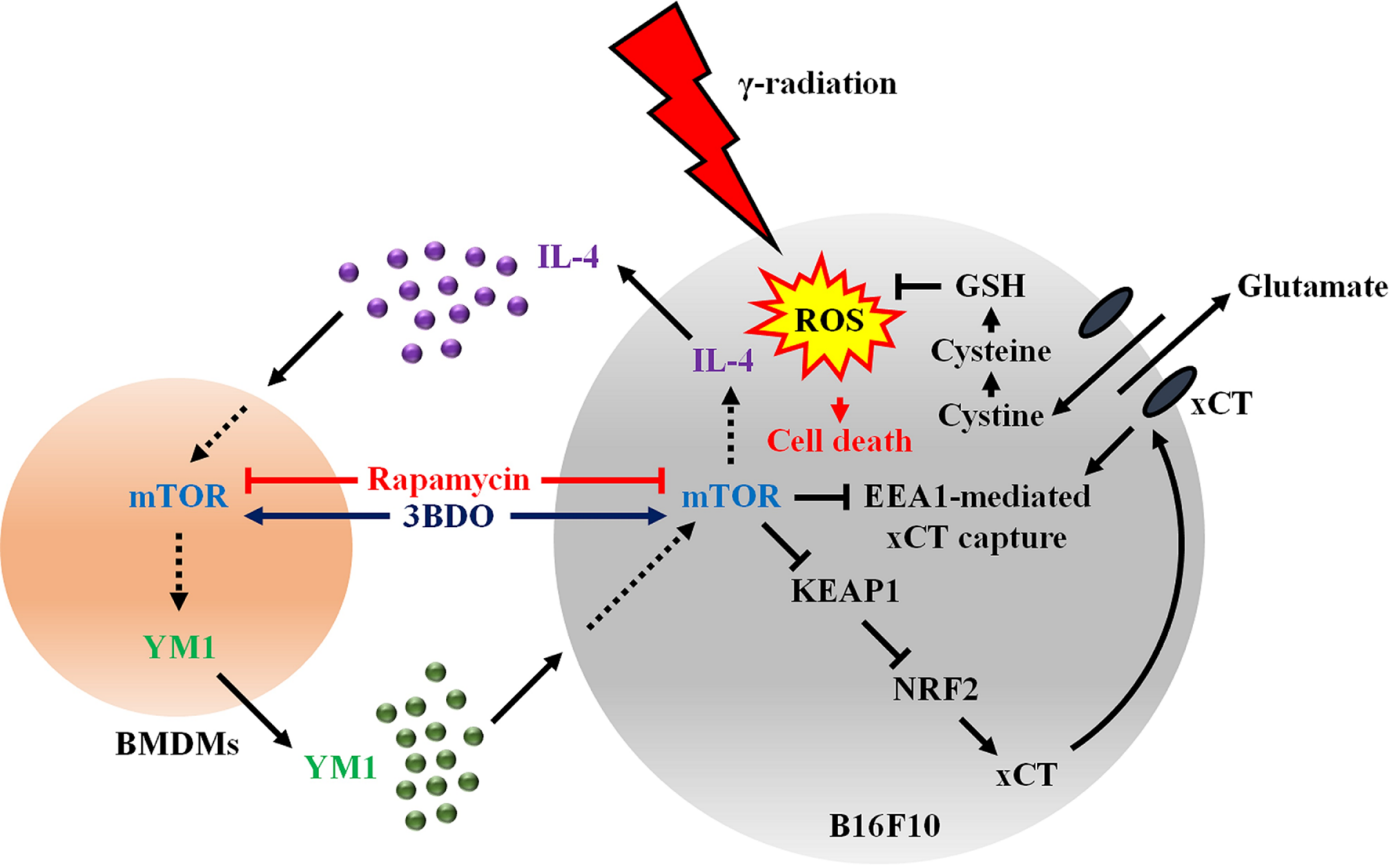
Activated mTOR signaling induces the secretion of IL-4 in melanomas and YM1 in macrophages in the tumor microenvironment. Secreted YM-1enhances the EEA1-mediated xCT capture *via* the KEAP1/NRF2 signaling pathway to promote antioxidant mechanisms through GSH in radiotherapy. Therefore, the regulation of mTOR activity using rapamycin could increase tumor sensitivity to γ-radiation-induced ROS by inhibiting the interaction between melanoma and macrophages.

YKL40 is the human homologue for mouse breast regression protein 39 (BRP39), but there is no corresponding homologue of human YKL39 in mice (14). In addition, YM1 is expressed only in mice (39). Therefore, the effect of YKL39 on human melanoma was evaluated, as there are no alternative CLPs for YM1 in humans. In this study, human recombinant YKL39 attenuated radiation-induced colocalization of xCT and EEA1 in Malme-3M, similar to mouse recombinant YM1. However, the effect of YKL39 on xCT expression was observed to be different depending on the cell line. Thus, further research is needed to determine whether YM1 and YKL39 attenuate EEA1-mediated xCT capture *via* the same mechanism in various tumor cases under γ-radiation. Our study shows that mTOR inhibition in the tumor microenvironment can attenuate the interaction between tumor cells and macrophages during radiotherapy (**Figure 8**). This study also provides a new strategy to enhance tumor radiosensitivity by blocking the system of maintaining ROS homeostasis in tumors.

## Data Availability Statement

The original contributions presented in the study are included in the article/**Supplementary Material**. Further inquiries can be directed to the corresponding author.

## Ethics Statement

The animal experiments in the present study were approved and confirmed by the Ethical Guidelines for Animal Experiments of Kangwon National University (KW-181214-1).

## Author Contributions

YW and Y-JJ designed the study. YW performed and analyzed the *in vivo* and *in vitro* experiments together with H-JL, JK, SK, SM, YJ, and JH contributed materials and analysis tools. YW and Y-JJ wrote the paper with input from the other authors. All authors contributed to the article and approved the submitted version.

## Funding

This research was supported by a grant from the Basic Science Research Program through the National Research Foundation of Korea (NRF), which is funded by the Ministry of Science, ICT and Future Planning of the Republic of Korea (2018R1D1A1B07049097 and 2021R1A2C1004525), and the Korea Basic Science Institute (National Research Facilities and Equipment Center) grant funded by the Ministry of Education (2020R1A6C101A195).

## Conflict of Interest

The authors declare that the research was conducted in the absence of any commercial or financial relationships that could be construed as a potential conflict of interest.
