# Supplementary material for: Rapamycin Promotes ROS-Mediated Cell Death via Functional Inhibition of xCT Expression in Melanoma Under γ-Irradiation
Source: Front Oncol. 2021 Apr 20;11:665420. doi: 10.3389/fonc.2021.665420 (PMC8093631; doi:10.3389/fonc.2021.665420)
Supplement: Supplementary file 1 [file DataSheet_1.docx]

Supplementary Material

# Supplementary Materials and Methods

**Reverse transcription PCR**

Total RNA was isolated using an RNeasy mini kit (QIAGEN, Valencia, CA, USA). Reverse transcription was performed with 2 μg of RNA. cDNA was amplified using the following primers (Geno Tech Ltd, Daejeon Korea):

mouse *nos2* FW: 5’-CCCTTCCGAAGTTTCTGGCAGCAGC-3’

mouse *nos2* Rv: 5’-GGCTGTCAGAGCCTCGTGGCTTTGG-3’ (Lee et al., 2009)

mouse *cxcl10* FW: 5’-CACCATGAACCCAAGTGCTGCCGT-3’

mouse *cxcl10* Rv: 5’-AGGAGCCCTTTTAGACCTTTTTTG-3’ (Yang et al., 2013)

mouse *fizz1* FW: 5’-GGTCCCAGTGAATACTGATGAGACCATAGA-3’

mouse *fizz1* Rv: 5’-CACCTCTTCACTCTTAGGACAGTTGGCAGC-3’ (Gangadharan et al., 2008)

mouse *pparγ* FW: 5’-CGTGATGGAAGACCACTCGC-3’

mouse *pparγ* Rv: 5’-AACCTGATGGCATTGTGAGA-3’ (Yu et al., 2011)

mouse *ccl22* FW: 5’-CCTGGTGGCTCTCGTCCTTC-3’

mouse *ccl22* Rv: 5’-CAGGGGATGGAGGTGAGTAA-3’ (Zheng et al., 2009)

PCR amplification was carried out for 35 cycles (annealing: 54°C) using Taq polymerase (Enzynomics; Daejeon, Korea), and then the PCR products were analyzed on 2% agarose gels with ethidium bromide staining.

**Antibodies and fluorescent staining reagents**

The following antibodies and staining reagents were used for western blotting, flow cytometry, and confocal microscopy: anti-caspase 3 (Cat# 9661), anti-LC3 (Cat# 2775), anti-beclin1 (Cat# 3738), anti-Atg12 (Cat# 4180), anti-Bcl2 (Cat# 3498), anti-Bax (Cat# 2772), anti-NOS2 (Cat# 13120), anti-phospho-p70 S6K (Cat# 9208), anti-PARP (Cat# 9542), anti-TIMP2 (Cat# 5738), anti-mouse (Cat# 7076), anti-rabbit (Cat# 7074), and propidium iodide (PI) (Cat# 4087), and they were obtained from Cell Signaling Technology (Beverly, MA, USA). Anti-CD11b (Cat# 101205), anti-CD206 (Cat# 141703), and anti-Class II MHC (Cat# 107606) were purchased from BioLegend (San Diego, CA, USA). Anti-YM-1 (Cat# ab93034), anti-Fizz1 (Cat# ab39626), anti-xCT (Cat# ab37185), anti-COX2 (Cat# ab52237), anti-MMP2 (Cat# ab37150), anti-MMP9 (Cat# ab38898), and anti-Keap1 (Cat# ab119403) were purchased from Abcam (Cambridge, MA, USA). Anti-p70 S6K (Cat# sc-8418), anti-Arginase-1 (Cat# sc-47715), anti-PPAR-γ (Cat# sc-7273), and anti-β-actin (Cat# sc-47778) were obtained from Santa Cruz Biotechnology (Santa Cruz, CA, USA). Anti-Ki67 (Cat# 14-5698-80), anti-phospho-NRF2 (Cat# PA5-67520), anti-NRF2 (Cat# PA5-68817), and DAPI (Cat# D3571) were purchased from Thermo Fisher Scientific (Rockford, IL, USA). In addition, CellROX Green Reagent (C10444) was also obtained from Thermo Fisher Scientific (Waltham, MA, USA). Anti-rabbit FITC (Cat# 111-095-003) and anti-rabbit TRITC (Cat# 715-025-150) were obtained from Jackson ImmunoResearch Labs (West Grove, PA, USA). Anti-IL-4 (Cat# 500-P54) was purchased from PeproTech (Rocky Hill, NJ, USA). Anti-EEA1 (Cat# NBP1-30914) was obtained from Novus Biologicals (Littleton, CO, USA).

**Correlation analysis of target genes in human patients**

RNA expression values of xCT, KEAP1, NRF2, and YKL39 were obtained from TCGA (PanCancer Atlas, 448 samples). TCGA datasets of skin cutaneous melanoma were examined using the cBioPortal (www.cbioportal.org) (Cerami et al., 2012; Gao et al., 2013). Gene expressions in melanoma patients was represented as RSEM (batch normalized from Illumina HiSeq_RNASeqV2; z-score threshold ± 2.0). Correlation plots of each mRNA expression level were analyzed within the website.

**Real-time metabolism assay**

An XFp Extracellular Flux Analyzer (Seahorse Bioscience, Billerica, MA, USA) was used for real-time analysis of a glycolytic rate assay (GRA) and the oxygen consumption rate (OCR). The measurements of the GRA and OCR were performed according to the manufacturer's instructions. Briefly, BMDMs that were variously stimulated were reseeded in Seahorse miniplates (5×10^4^ cells/well) coated with Cell-Tak (Corning Life Sciences, Acton, MA, USA) and then incubated at 37℃ in a non-CO_2_ incubator for 50 min. For GRA analysis, cells were sequentially treated with rotenone plus antimycin A (1.25 μM/well for each, Seahorse Bioscience) and 2-deoxy-D-glucose (50 mM/well, Seahorse Bioscience) for 70 min. OCR measurement was performed through sequential treatment with oligomycin (1.5 μM/well, Seahorse Bioscience), carbonyl cyanide-4-(trifluoromethoxy)phenylhydrazine (FCCP, Seahorse Bioscience, 1.5 μM/well) and rotenone (1.25 μM/well, Seahorse Bioscience) for 80 min. The GRA and OCR were recorded according to the manufacturer's instructions.

**Statistical analysis**

All data are presented as the mean ± SD. Statistical analyses were performed using one-way or two-way analysis of variance (ANOVA) with a Bonferroni correction by using GraphPad PRISM software. P values < 0.05 were considered significant. Experiments were repeated a minimum of 3 times for each condition.

# 1.2. Supplementary Figures


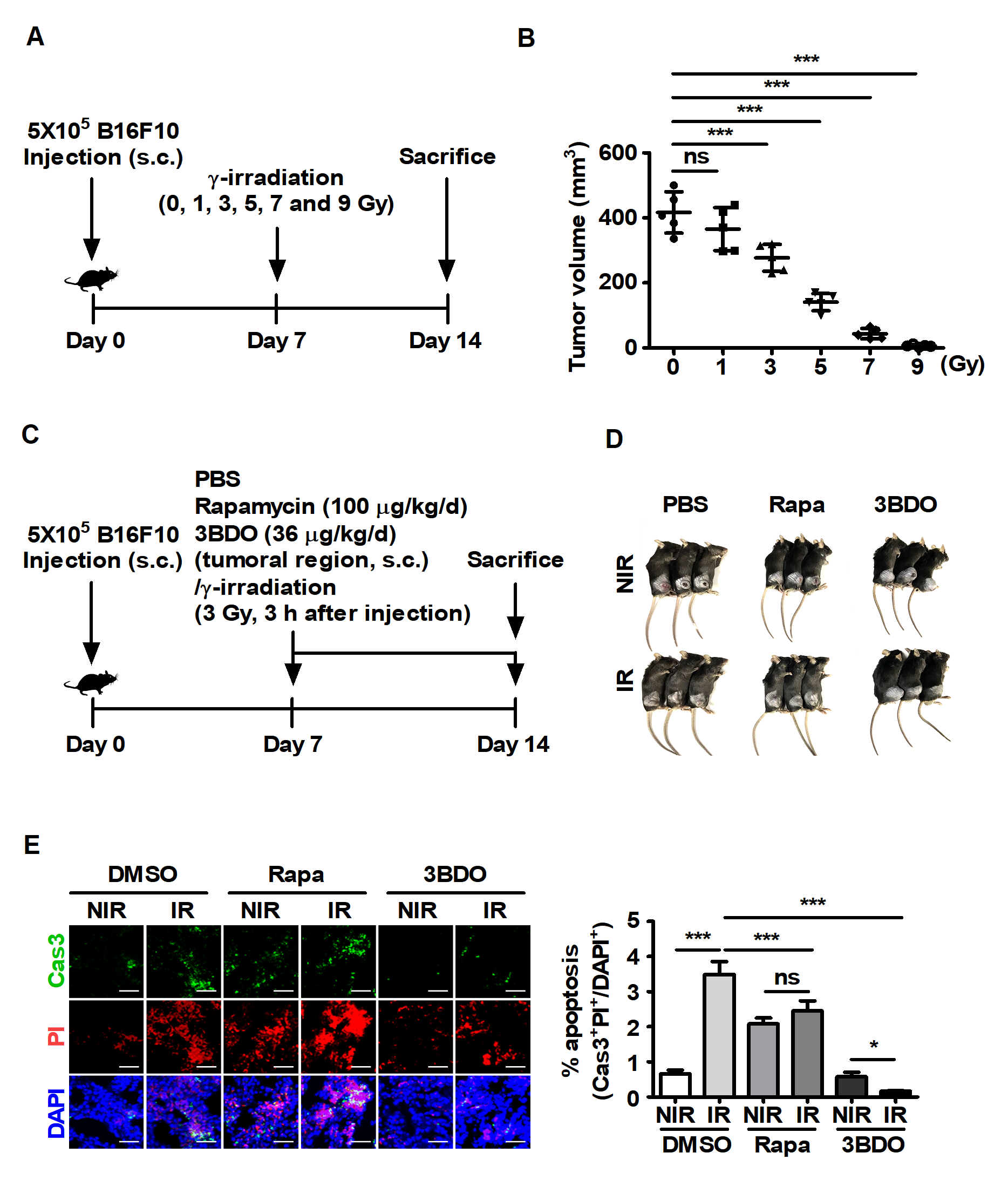


**Supplementary Figure 1.** Pharmaceutical regulation of mTOR activation affects B16F10 tumor development. **A.** A schematic representation of the in vivo experimental design for radiation dose determination is shown. **B.** The volumes of tumors established with the protocol shown in A were measured with calipers (n=5/group). **C.** A schematic representation of the in vivo experimental design for the evaluation of mTOR activation in tumor development is shown. **D.** All images were of mice treated as shown in **C, E.** Intratumoral levels of caspase-3 (green) and PI (red) were analyzed by confocal microscopy. All images were quantified using ImageJ. Scale bars, 100 μm. The bars and error bars represent the mean ± SD; *, *P* < 0.05; **, *P* < 0.01; ***, *P* < 0.001; ns, not significant.


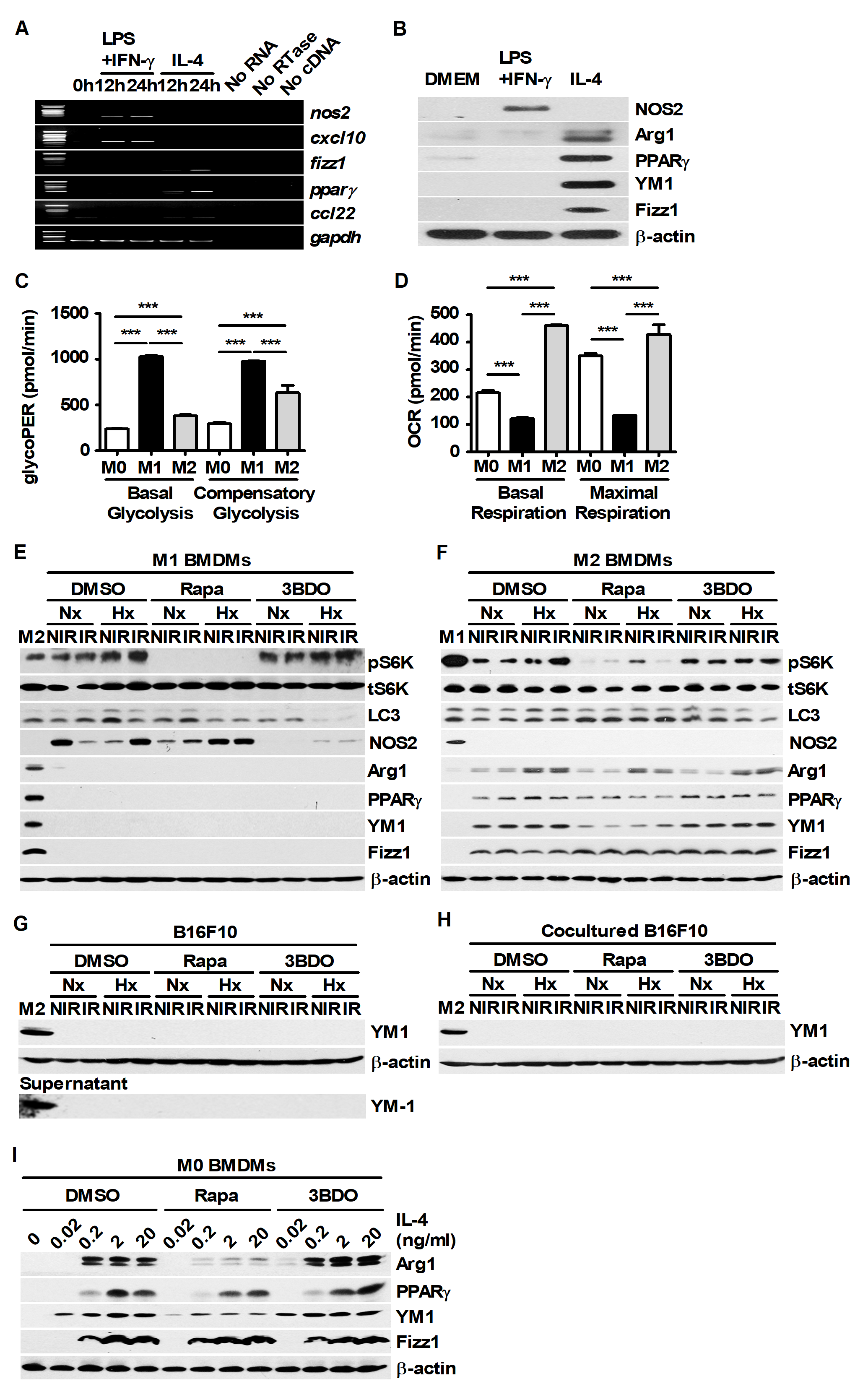


**Supplementary Figure 2.** Pharmaceutical regulation of mTOR activation affects polarized macrophages. a and b, Bone marrow cells from C57BL/6 mice were incubated with DMEM containing 20% FBS and 30% L929-conditioned medium for 5 days. Fully differentiated BMDMs were treated with 100 ng/ml LPS plus 20 ng/ml IFN-γ or 20 ng/ml IL-4 for 24 h. **A.** mRNA levels of nos2, cxcl10, fizz-1, ppar-γ, ccl2, and gapdh in polarized BMDMs were analyzed by RT-PCR. **B.** The protein levels of NOS2, Arginase1, PPARγ, YM1, Fizz1, and β-actin were detected by western blotting. **C and D.** BMDMs were treated with 100 ng/ml LPS plus 20 ng/ml IFN-γ or 20 ng/ml IL-4 for 24 h. After macrophage polarization, the BMDMs were incubated under the indicated conditions for 24 h, reseeded in a Cell-TAK-coated XF plate and then incubated in XF base medium at 37°C, in a non-CO_2_ chamber for 50 min. Glycolytic proton efflux rate (glycoPER) and the oxygen consumption rate (OCR) in the BMDMs were assessed by a Seahorse assay. **E and F.** M1 and M2 macrophages were incubated in the indicated conditions for 24 h. The protein levels of p70 S6K, phospho-p70 S6K, LC3, NOS2, arginase1, PPARγ, YM1, and Fizz1 in BMDMs were evaluated by western blotting. β-actin was used as an internal control. **G and H**, Single- cultured (**G**) and BMDM-cocultured B16F10 cells (**H**) were incubated in the indicated conditions for 24 h. Intracellular- and secreted- YM1 levels in B16F10 cell cultures were detected by western blotting. **I.** BMDMs were stimulated with the indicated concentrations of recombinant mouse IL-4 for 24 h. The protein levels of Arginase1, PPARγ, YM1, Fizz1, and β-actin in the BMDMs were evaluated by western blotting. The bars and error bars represent the mean ± SD; *, *P* < 0.05; **, *P* < 0.01; ***, *P* < 0.001; ns, not significant.

**
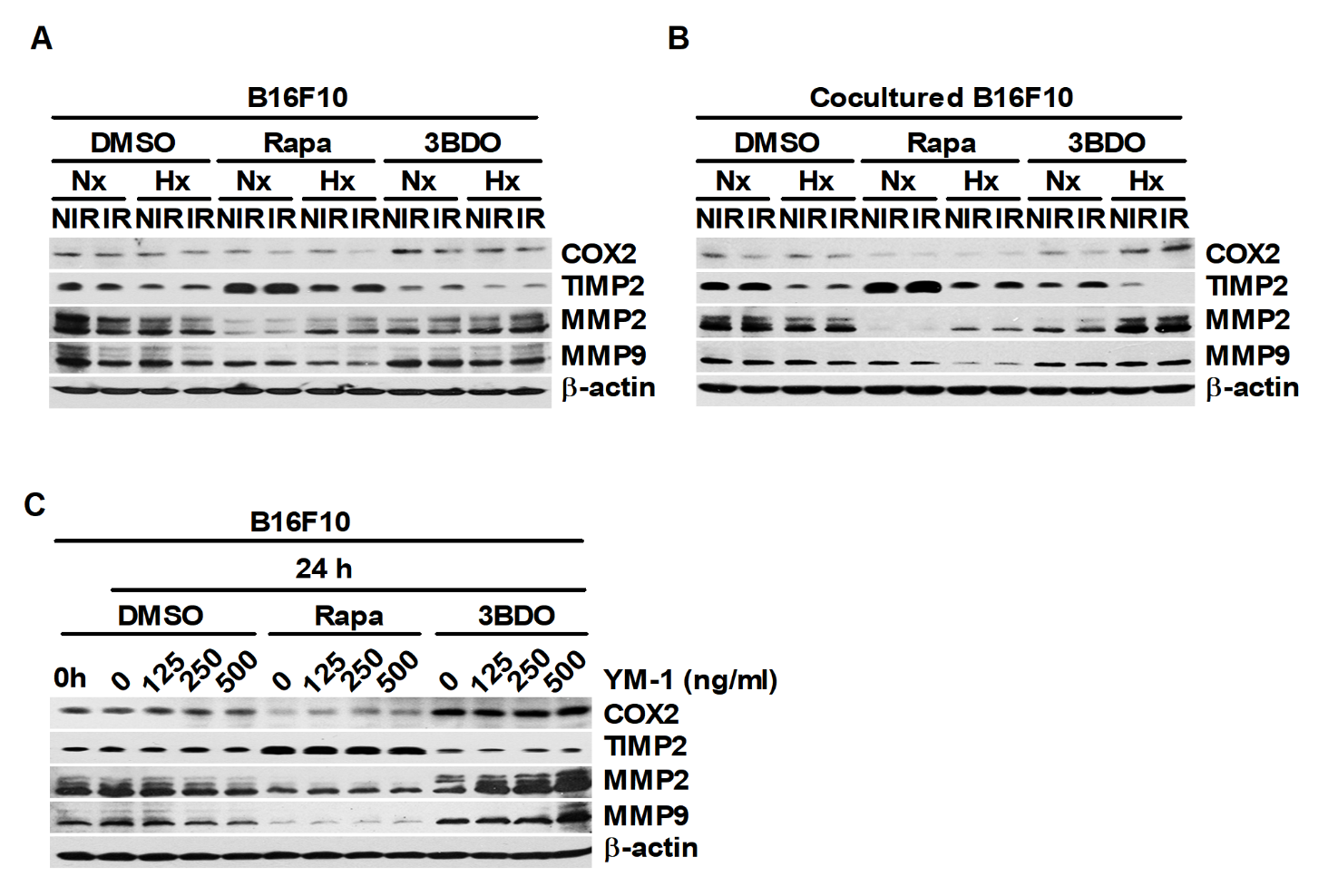
**

**Supplementary Figure 3.** Recombinant YM-1 does not affect the expression of EMT-related proteins in B16F10 cells. **A and B.** B16F10 cells cultured alone or cocultured with BMDM were treated with DMSO, 100 nM rapamycin or 100 nM 3BDO under the indicated conditions. Intracellular levels of COX2, TIMP2, MMP2, MMP9, and β-actin in B16F10 cells were evaluated by western blot. **C.** B16F10 were treated with DMSO, 100 nM rapamycin or 100 nM 3BDO for 3 h and then stimulated with the indicated concentrations of recombinant mouse YM-1 for 21 h. The expression levels of COX2, TIMP2, MMP2, MMP9, and β-actin in B16F10 cells were detected by western blotting.


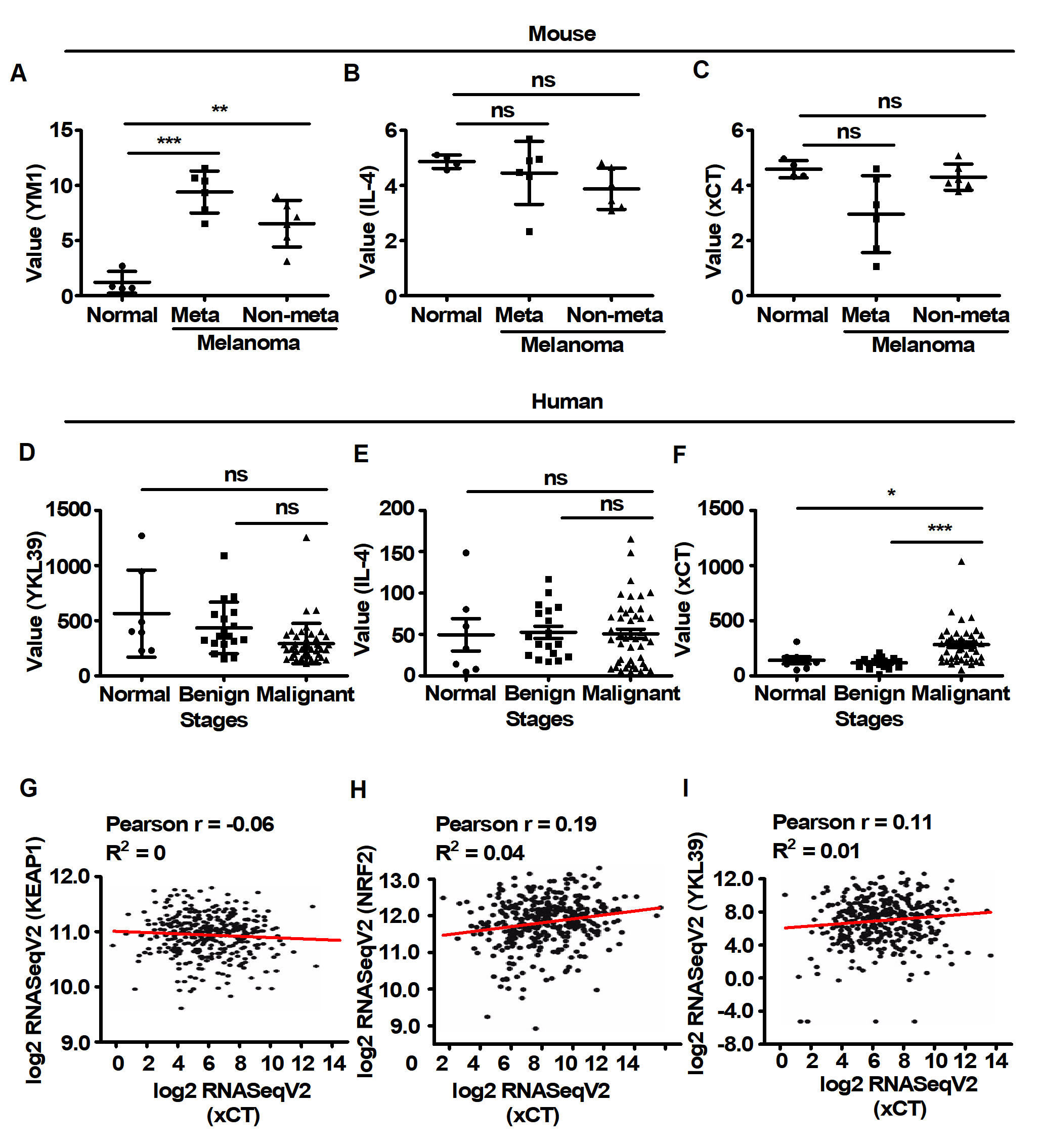


**Supplementary Figure 4.** The expressions of YM1 in mouse and xCT in human are prominent in melanoma. Human YKL39 affects γ-ray-induced colocalization of xCT and EEA1 in Malme-3M, a human melanoma cell line. **A-F.** Scatter-plots for the mRNA values of mouse YM1 (**A**), mouse IL-4 (**B**), mouse xCT (**C**), human YKL39 (**D**), human IL-4 (**E**), and human xCT (**F**) in the GEO database set (**A-C**: GSE29074; **D-F**: GSE3189) are presented. **G-I.** Correlation plots between mRNA values were obtained from cBioPortal. All data were assessed by GraphPad Prism. The bars and error bars represent the mean ± SD; *, *P* < 0.05; **, *P* < 0.01; ***, *P* < 0.001; ns, not significant.

**References**

Cerami, E., Gao, J., Dogrusoz, U., Gross, B.E., Sumer, S.O., Aksoy, B.A., et al. (2012). The cBio cancer genomics portal: an open platform for exploring multidimensional cancer genomics data. *Cancer Discov* 2(5)**,** 401-404. doi: 10.1158/2159-8290.CD-12-0095.

Gangadharan, B., Hoeve, M.A., Allen, J.E., Ebrahimi, B., Rhind, S.M., Dutia, B.M., et al. (2008). Murine gammaherpesvirus-induced fibrosis is associated with the development of alternatively activated macrophages. *J Leukoc Biol* 84(1)**,** 50-58. doi: 10.1189/jlb.0507270.

Gao, J., Aksoy, B.A., Dogrusoz, U., Dresdner, G., Gross, B., Sumer, S.O., et al. (2013). Integrative analysis of complex cancer genomics and clinical profiles using the cBioPortal. *Sci Signal* 6(269)**,** pl1. doi: 10.1126/scisignal.2004088.

Lee, J.A., Song, H.Y., Ju, S.M., Lee, S.J., Kwon, H.J., Eum, W.S., et al. (2009). Differential regulation of inducible nitric oxide synthase and cyclooxygenase-2 expression by superoxide dismutase in lipopolysaccharide stimulated RAW 264.7 cells. *Exp Mol Med* 41(9)**,** 629-637. doi: 10.3858/emm.2009.41.9.069.

Yang, C.S., Kim, J.J., Lee, S.J., Hwang, J.H., Lee, C.H., Lee, M.S., et al. (2013). TLR3-triggered reactive oxygen species contribute to inflammatory responses by activating signal transducer and activator of transcription-1. *J Immunol* 190(12)**,** 6368-6377. doi: 10.4049/jimmunol.1202574.

Yu, W.G., Xu, G., Ren, G.J., Xu, X., Yuan, H.Q., Qi, X.L., et al. (2011). Preventive action of curcumin in experimental acute pancreatitis in mouse. *Indian J Med Res* 134(5)**,** 717-724. doi: 10.4103/0971-5916.91009.

Zheng, T., Oh, M.H., Oh, S.Y., Schroeder, J.T., Glick, A.B., and Zhu, Z. (2009). Transgenic expression of interleukin-13 in the skin induces a pruritic dermatitis and skin remodeling. *J Invest Dermatol* 129(3)**,** 742-751. doi: 10.1038/jid.2008.295.
